# Role of Viral Infections in the Pathogenesis of Sjögren’s Syndrome: Different Characteristics of Epstein-Barr Virus and HTLV-1

**DOI:** 10.3390/jcm9051459

**Published:** 2020-05-13

**Authors:** Hideki Nakamura, Toshimasa Shimizu, Atsushi Kawakami

**Affiliations:** Department of Immunology and Rheumatology, Unit of Advanced Preventive Medical Sciences, Division of Advanced Preventive Medical Sciences, Nagasaki University Graduate School of Biomedical Sciences, Nagasaki 852-8501, Japan; toshimasashimizu2000@yahoo.co.jp (T.S.); atsushik@nagasaki-u.ac.jp (A.K.)

**Keywords:** viral infection, Epstein-Barr virus, HTLV-1, salivary gland epithelial cell

## Abstract

Viruses are possible pathogenic agents in several autoimmune diseases. Sjögren’s syndrome (SS), which involves exocrine dysfunction and the appearance of autoantibodies, shows salivary gland- and lacrimal gland-oriented clinical features. Epstein-Barr virus (EBV) is the most investigated pathogen as a candidate that directly induces the phenotype found in SS. The reactivation of the virus with various stimuli induced a dysregulated form of EBV that has the potential to infect SS-specific B cells and plasma cells that are closely associated with the function of an ectopic lymphoid structure that contains a germinal center (GC) in the salivary glands of individuals with SS. The involvement of human T-cell leukemia virus type 1 (HTLV-1) in SS has been epidemiologically established, but the disease concept of HTLV-1-associated SS remains unexplained due to limited evidence from basic research. Unlike the cell-to-cell contact between lymphocytes, biofilm-like structures are candidates as the mode of HTLV-1 infection of salivary gland epithelial cells (SGECs). HTLV-1 can infect SGECs with enhanced levels of inflammatory cytokines and chemokines that are secreted from SGECs. Regardless of the different targets that viruses have with respect to affinitive lymphocytes, viruses are involved in the formation of pathological alterations with immunological modifications in SS.

## 1. Introduction

The pathogenesis of Sjögren’s syndrome (SS), which affects salivary glands (SGs) as well as lacrimal glands (LGs), involves multiple factors including genetic elements [1,2,3] and subsequent environmental factors [4]. The clinical characteristics of individuals with SS are exocrine dysfunction (e.g., xerostomia and xerophthalmia) with the appearance of autoantibodies (including anti-Ro/SS-A and La/SS-B antibodies) [5]. With respect to genetic elements of SS, human leukocyte antigen (HLA) alleles have shown the strongest association with SS, and significant variations in HLA alleles by ethnicity have been revealed. Environmental factors that affect gender differences or the production of various autoantibodies may exert estrogen deficiency-mediated immunological effects [6] or alterations of the oral microbiome [7].

In addition, viral infections are profoundly associated with the activation of innate immunity, followed by an acquired immune response. Recognition of single-stranded ribonucleic acid (RNA) by toll-like captor 7/9 was a representative sensor of innate immunity in SS [8,9]. With the activation of an innate immunity signal with an interferon-gamma (IFN-γ) signature in viruses, the subsequent function of antigen presentation through the induction of major histocompatibility complex (MHC) is followed by an expansion of antigen-specific autoreactive T cells. In contrast, some viruses have a unique characteristic, i.e., a so-called in vivo ‘reactivation’ by various stimuli up to years after the initial infection [10]. In this review, we provide a comprehensive explanation of the roles of viruses that cause a breakdown of immune tolerance or modulation of the immune system in the pathogenesis of SS, and we describe the various detection analyses that have been used for these viruses. We focus on the actions of Epstein-Barr virus (EBV) and retroviruses by comparing the effects of these viruses in salivary glands of individuals with Sjögren’s syndrome, as well as on the epidemiological and immunological findings.

## 2. Viral Infection and Autoimmune Diseases (AIDs)

### 2.1. Possible Mechanism Triggered by Viral Infection in AIDs

An acute or chronic viral infection is thought to play a crucial role as a triggering phase of the immunological instability that is required for the formulation of the chronic inflammatory condition in AIDs. As an initial step of innate immunity that involves several functions of cytotoxic T lymphocytes or natural killer T cells, the recognition of mobilized pathogens by receptors of innate immunity is required. Pathogen-associated molecular patterns (PAMPs) are common structures detected in pathogenic microbes that contribute to the initiation of innate immunity [11]. In concert with the detection of PAMPs, the concept of pattern recognition receptors (PRRs) was defined; PRRs have several molecules including toll-like receptors (TLRs), C-type lectin receptors, nucleotide-binding oligomerization domain (NOD)-like receptors (NLRs), and retinoic acid inducible gene-I (RIG-I)-like receptors (RLRs) [12,13,14]. Toll-like receptor 7 (TLR7) in particular is known to recognize single-stranded RNA viruses in exosomes of plasmatoid dendritic cells (pDCs) followed by a MyD88-dependent activation of the nuclear factor kappa B pathway after the assembly of tumor necrosis factor (TNF)-receptor associated factor-6 or interferon (IFN)-regulatory factor-7 (IRF-7) [15], which are associated with the induction of type I IFNs.

In our previous study, the expression of downstream signal transduction after stimulation with the TLR7 ligand was confirmed in the salivary glands of patients with SS and in an in vitro investigation using cultured epithelial cells obtained from SS patients [8], and pDCs appeared to be a source of type I IFN (including IFN-α and IFN-β). Regarding the close association between retinoic acid-inducible gene I (RIG-I)-like family signaling and SS, Maria et al. [16] demonstrated that RIG-I and melanoma differentiation associated gene-5 (MDA-5) were strongly expressed in mononuclear cells (MNCs) of salivary glands from SS patients. They also demonstrated that TLR7 stimulation had the potential to activate the signaling pathway of RIG-I-like family members in vitro. These observations suggested that TLRs and RLRs that were stimulated in salivary gland pDCs or monocytes are closely associated with RNA-sensing receptor-mediated innate immunity with the presentation of the type I IFN signature.

### 2.2. The Relationship between Autoimmune Conditions and Viral Infection

A relationship between typical AIDs and viral infection has been clarified over the past three decades. With respect to outbreaks of type 1 diabetes mellitus, the involvement of coxsackie B virus (an enterovirus) has been suggested, but the epidemiological evidence remains controversial regardless of a systematic review of 26 case-control studies [17]. In contrast, in a clinical study [18] using autopsied specimen RIG-I and MDA-5, type I IFNs were also strongly expressed in cells of enterovirus-infected pancreas in patients with fulminant type 1 diabetes mellitus.

Regarding the relationship between pathogens and the axonal form of Guillain-Barré Syndrome (GBS) with the appearance of antibodies against GM1 or GD1b, the involvement of Campylobacter infection as an exposure to bacteria has been discussed [19], and the involvement of Campylobacter toward GBS from a molecular biological perspective has been clarified. In addition, Zika virus infections in Columbia [20] were reported to be associated with GBS accompanied by a high degree of cranial nerve involvement or autonomic dysfunction.

Concerning connective tissue diseases, there are debates with respect to the relationship between systemic lupus erythematosus (SLE) and some viruses [21], and the involvement of parvovirus and EBV was discussed in serological research. It was reported that 3.9% of sera from SLE patients had a viral genome toward parvovirus, and among these patients, those with secondary anti-phospholipid syndrome (APS) had antibodies against parvovirus [22], suggesting a close association between APS-related antibody production and parvovirus. Regarding the relevance of the association between EBV and pathogenesis in SLE, the impairment of EBV-specific CD8+ T cells was reported to be associated with the activation of B cells in SLE patients who had high EBV viral loads [23]. The involvement of viral infection in rheumatoid arthritis (RA) has also been suspected for many years, but there is no definitive evidence regarding this matter. In a Norwegian study [24], there were no differences between patients with RA and healthy controls with respect to IgG antibodies against EBV and parvovirus B19 in sera.

Human T-cell leukemia virus type 1 (HTLV-1) has been vigorously investigated because HTLV-1 transgenic mice showed chronic erosive arthritis that resembled the synovitis observed in RA [25,26]. However, evidence that HTLV-1 infection directly induces typical RA has not yet been obtained in human studies, although the expression of HTLV-1-related antigen was demonstrated in synovial tissue obtained from RA patients [27]. Rather, there is concern regarding responsiveness to molecular targeted drugs including biologics (such as TNF inhibitors) among HTLV-1-seropositive patients with RA when therapeutic strategies are being considered [28,29]. No change of viral load or clonality was observed in HTLV-1-seropositive RA patients who were treated with a TNF inhibitor [28], and the efficacy of a TNF inhibitor was attenuated in HTLV-1-seropositive RA patients, especially in those who were anti-citrullinated protein antibody-positive [29]. For the treatment of HTLV-1-seropositive RA patients in endemic areas, the molecular targeted drugs’ response rates and tumorigenesis remain serious concerns.

## 3. EBV Infection and Sjögren’s Syndrome

### 3.1. Characteristics of the Infection Mechanism in EBV

EBV is a member of the family of herpes viruses. It is a double-stranded DNA virus, and the nomenclature of EBV is human herpes virus 4 (HHV-4). EBV was originally isolated from culture medium of Burkitt tumor cells [30], and EBV was an etiological agent for infectious mononucleosis or nasopharyngeal carcinoma in China [31]. Clinical issues of concern are the activation of EBV in specific conditions; for example, the reactivation of EBV was observed in the early infection phase of human immunodeficiency virus (HIV), and EBV reactivation within six months was observed before HIV seroconversion [32]. The reactivation of EBV has also been frequently detected under immunosuppressive conditions. The management of EBV reactivation in cases of stem cell transplantation under intensive immunosuppression is a serious issue [33].

In the field of rheumatology, the involvement of EBV in methotrexate (MTX)-associated lymphoproliferative disorder (MTX-LPD) is a considerable problem in the era of molecular-targeted therapy for RA [34]. In addition, chronic active EBV (CAEBV) infection [35] was classified as a new entity in the revised version of World Health Organization (WHO) criteria, in which EBV can infect not only B cells but also T cells and natural killer cells, resulting in a poor prognosis. There is a specific mode of infection in EBV that includes a latent and lytic infection phase during the EBV life cycle [36]. The latent infection of EBV was usually observed in peripheral blood B cells with the expression of latent membrane protein (LMP), which is associated with B-cell transformation [37]. In addition, the latency of EBV was categorized into four patterns (from 0 to III) based on the host cells’ condition [38] with various expression patterns of EB nuclear antigen (EBNA)1 or EB-encoding regions (EBERs). In the latent phase, viral particles are not produced with the expression of non-structural protein in B cells. In contrast, the latent phase was switched to the lytic phase by LMP1 that was induced by BRLF1 [39], and once EBV had entered the lytic cycle, emitted EBV virions infected mucosal epithelial cells of the pharynx or ductal epithelial cells of salivary glands.

### 3.2. Chronological Changes in the Interpretations of EBV Infection in SS

With respect to the relationship between SS and EBV infection, our PubMed search identified 149 reports, with many conflicting results and opinions regarding SS per se and EBV-associated hematological tumors. Representative studies about the detection of EBV in SS are listed in Table 1.

A first definitive study [40] regarding EBV in Sjögren’s syndrome was conducted in 41 patients with RA; IgG antibody against EBV capsid antigen (EBVCA) was detected in all 41 RA patients, 26 patients with sicca syndrome, and 26 healthy subjects. Subsequently, Fox et al. [41] reported that EBV DNA revealed by slot hybridization was detected in 8 of 20 parotid saliva samples from SS patients, whereas EBV DNA was not detected in saliva from control subjects. Fox et al. also reported positive cytoplasmic staining of EBV-encoded early antigen (EA-D) in 8 of 14 salivary gland samples from SS patients. Whittingham et al. [54] hypothesized that EBV is an etiological agent of SS (especially for early-stage SS) in light of their observation of the binding of anti-La/SS-B antibody to viral RNAs or molecules encoded by EBV.

It was reported that, compared to control subjects, the titer of anti-ENBA antibodies but not that of anti-cytomegalovirus (CMV) antibodies was higher in sera from SS patients, and anti-EBNA antibodies were dominantly detected in anti-La/SS-B antibody-positive patients with SS [55]. Venables et al. subsequently reported that EBV DNA was present in the salivary gland biopsy specimen of 6 of 10 control subjects (60%) compared to 17% in the salivary gland biopsy specimens of SS patients [44]. They also detected EBV DNA in the salivary glands of five control subjects without HLA-class II antigen, suggesting that EBV infection was not specific for SS. With respect to the tumorigenicity of EBV, Fox et al. used a restriction fragment length polymorphism technique and reported that EBV or HHV-6 was associated with the onset of lymphomas [56]. In a study applying probes for both EBV and HHV-6 in cultured salivary gland epithelial cells (SGECs), only HHV-6 was detected, but HHV-6 was not related to SS [46].

Because benign lymphoepithelial lesions (BLELs) are unique pathological features in SS, Di Giuseppe et al. examined the involvement of EBV in BLELs [57]: they detected no EBER1 expression in salivary glands from SS patients and suggested that there is no involvement of latent EBV infection in SS. Regarding the relationship between CMV and EBV, their DNA in labial salivary glands (LSGs) from SS patients and subjects with non-specific sialadenitis was examined by polymerase chain reaction (PCR), which revealed no significant difference between the two patient groups [58]. Di Giuseppe et al. also contended that the persistence of these two viruses after the initial infection in LSGs was reasonable, but a direct involvement of EBV in the pathogenesis of SS was not suggested by their findings. Other investigators detected EBV DNA by in situ hybridization (ISH) and PCR in 19% of their SS patients versus 3% of the control subjects [49]. In contrast, EBV LMP was identified in 17% of their SS patients and 22% of the control subjects, indicating that the involvement of EBV can be influenced by the presence of undetermined factors.

Regarding the involvement of EBV in the pathogenesis of lymphomas, Royer et al. examined the occurrence of non-Hodgkin’s lymphoma (NHL) and they identified cases with low-grade marginal zone lymphoma (MZL) and mucosa-associated lymphoid tissue (MALT) lymphoma; however, no obvious association between these SS and EBV infection was observed based on the presence of LMP proteins and EBER RNA [59]. In addition, the clinical implications of EBV in relation to the frequency of the detection of EBV DNA and related proteins have not been established. Regarding the association between the disease activity of SS and EBV infection, it was first demonstrated that significant levels of anti-EA-D antibodies were present in SS patients with articular manifestations described by items of the EULAR Sjögren’s Syndrome Disease Activity Index (ESSDAI) [52]. Croia et al. took ectopic lymphoid structures (ELSs) into account, considering the selectivity of active EBV infection in SS as described below in the Pathogenesis section (Section 3.4) [53]. Regarding lymphoma observed in SS, it was demonstrated that among 382 patients with SS, 2.6% (*n* = 10) were diagnosed with EBV-associated follicular lymphoma, and 8 of these 10 patients showed a positive expression of LMP1 [60].

### 3.3. The Reactivation and Detection of EBV in SS

The word ‘reactivation’ in SS was first used in the 1980s by researchers examining the reactivity of monoclonal antibodies against EBV in salivary glands of individuals with SS [61,62]. Since then, the interpretation of the concept of reactivation has changed, as have the techniques for detecting EBV DNA and proteins. Saito et al. [55] demonstrated the usefulness of a PCR method to detect and monitor EBV DNA in salivary gland epithelial cells and peripheral blood. They also highlighted the importance of the rapid detection of EBV reactivation under immunosuppressive conditions and in lymphoproliferative disorders. Mariette et al. [56] introduced a combination of ISH using BamH1-W fragment and PCR reaction to detect EBV DNA in salivary glands from SS patients and control subjects. They observed positivity predominantly in the specimens from the SS patients, but they reported that there was no evidence to show that EBV infection was directly involved in the destruction of the glandular structure.

ISH was later used to detect EBV-specific DNA in patients with secondary SS in which epithelial cells positive for EBV DNA were observed around areas with salivary gland destruction or lymphoepithelial lesions [63]. By using an enzyme-linked immunosorbent assay (ELISA) and a western blot analysis, Inoue et al. then observed high IgG antibody titers against EBNA antigens in sera from SS patients compared to sera from normal subjects [46]. With respect to the reactivation of EBV, Saito et al. used reverse transcriptase PCR coupled with PCR and immunohistochemistry (IHC), which revealed a strong expression of thioredoxin (TRX) in infiltrating B cells and epithelial cells in salivary glands from most of their SS patients [64]. In addition, an anatomical association between TRX and EBV as well as the co-expression of TRX message and EBV DNA were confirmed. In an in vitro experiment by those authors, B-cell lines that were infected with EBV frequently expressed TRX. These findings were the first to suggest the usefulness of detecting factors of EBV reactivation.

Regarding a link between tumorigenesis and EBV reactivation, increased reactivity was demonstrated toward EBV EA proteins such as BHRF1 (which is a viral homologue of Bcl-2 in rheumatic diseases including SS) [48]. A clear and frequent expression of interleukin (IL)-12 in infiltrating B cells and salivary gland tissue of SS patients was shown to correspond to EBV DNA [65], suggesting a relationship between EBV reactivation and Th1 cytokine. The involvement of the aryl hydrocarbon receptor (AhR, which binds to 2, 3, 7, 8-tetrachlorodibenzo-p-dioxin, or TCDD) in the reactivation of EBV was also reported [51]; that research group demonstrated an enhancement of BZLF1 transcription that mediated the switch to the lytic form, as well as a BZLF1 message and EBV DNA due to TCDD. These findings suggest that the ligation of AhR had the potential to induce the reactivation of EBV in both B cells and salivary epithelial cells.

### 3.4. EBV-Mediated Pathogenesis Observed in SS

Immunological and virological considerations have shaped the perspective on the involvement of EBV in the pathogenesis of Sjögren’s syndrome. Yamaoka et al. reported an increase in the proportion of polyclonal B cells in accord with the elevation of antiviral capsid antigen in SS [54]. Different findings were obtained by using a fusion protein (C28k) and synthetic peptides in an ELISA: there was no significant difference in the level of IgG antibodies in SS patients and healthy controls [66]. A counter-argument regarding these findings could be offered based on the differences in antigenic epitopes or the isolation of EBV from SS patients with additional in vitro observations.

Regarding the hypothesis of the direct involvement of EBV in SS, positive reactivity to EBV-related nucleo-cytoplasmic antigen was reported in 24 patients with SS, although the reactivity data differed between immunofluorescence and immunoblot analyses [67]. Correlations between aqueous tear deficiency and an elevated titer of EBV antigens as well as between SS and human leukocyte antigen (HLA)-DR+ CD8 lymphocytes were observed in lacrimal glands (LGs) [68], suggesting the presence of acquired immunological dysfunction in SS. With respect to the direct involvement of EBV in B-cell immunity, the production of EBV from a B-cell line that was newly established from peripheral blood mononuclear cells of patients with SS was demonstrated [47,69].

Alpha-fodrin was indicated as a possible autoantigen for SS [70], and Inoue et al. revealed that lymphoid cells that were reactivated by EBV had the potential to cleave alpha-fodrin as a 120-kDa fragment [50]. ZEBRA mRNA was shown to be a marker of the activation of the lytic cycle [71]. These findings showed novel functions that involved an apoptotic protease capability of reactivated EBV as an integrated concept in the pathogenesis of SS. Notably, the production of alpha-fodrin cleaved by EBV might be associated with antigen presentation, because 120-kDa alpha-fodrin was detected in sera from SS patients [70]; it may thus be involved in the pathogenesis of SS. A study regarding saliva as a soluble factor for EBV reactivation demonstrated that BZLF1 promotor was also activated in saliva under the presence of transforming growth factor-beta 1 (TGF-β1) induced by mitogen-activated protein kinase [72].

In the most recent development regarding the pathogenesis of SS, Croia et al. demonstrated SS-specific B-cell reactivation by EBV in their examination of ectopic lymphoid structures (ELSs) [53]. Although ELSs, which are an ectopic germinal center (GC) are known as a locus for B-cell activation and subsequent autoantibody production (as well as posing a risk for the development of lymphoma) [73,74], the affinity of EBV toward ELSs is a unique and crucial point. Croia et al. [53] described the following six important observations: (1) Lytic EBV infection was exclusively present in ELSs of salivary glands from patients with SS. (2) CD20^+^ B cells and CD138^+^ plasma cells around ELSs expressed EBER. (3) The expression of epithelial LMP was observed in both SS and nonspecific sialadenitis. (4) An antigen expressed during the lytic cycle, BFRF-positive CD138+ plasma cells that EBV lytically infected around ELSs, displayed Ro52. (5) SCID mice that received transplants of SS salivary gland tissue containing ELSs exhibited the ability to produce anti-Ro52/SS-A and anti-La/SS-B antibodies. (6) EBV that had increased affinity to ELSs in SS salivary glands was closely associated with impaired CD8^+^ T-cell cytotoxicity. These observations clearly demonstrated that the lytic phase of EBV has affinity to both ELSs and plasma cells with the capability to produce SS-specific autoantibodies.

## 4. HTLV-1 Infection and SS

### 4.1. Retrovirus Infection (Other Than HTLV-1 Infection) and SS

Representative studies on the involvement of retroviruses in SS are listed in Table 2.

The possibility of retrovirus infections other than HTLV-1 infection in individuals with SS was a research focus in the early 1990s. In 1989 it was reported that patients with HIV-associated salivary gland disease (HIV-SGD) showed a high frequency of parotid enlargement, sicca symptoms, and a low CD4/8 ratio in LSG biopsy specimens [75]. Interestingly, no anti-Ro/SS-A or La/SS-B antibodies were detected in the enrolled patients. Garry and colleagues described a human intracisternal A-type retroviral particle that was closely associated with HIV which they detected in lymphoid cells that were exposed to homogenates of salivary gland tissue from SS patients [76]. In investigations of the presence of serum antibodies of retrovirus, sera from 30% of healthy subjects had positive reactivity toward p24 (gag) but not gp41 or gp120 (env), with low reactivity to Ro/SS-A and La/SS-B [77,78]. Itescu et al. reported cases of HIV-positive patients demonstrating parotid enlargement, sicca symptoms, and CD8 T-lymphocyte dominance with HLA-DR5 [79], and the term ‘diffuse infiltrative lymphocytosis syndrome (DILS)’ was proposed [80].

The diagnostic criteria of DILS are as follows: (1) HIV infection with positive serology, (2) bilateral salivary gland enlargement or xerostomia, (3) persistence of signs or symptoms for ≥6 months, and (4) histologic confirmation of salivary or lacrimal gland lymphocytic infiltration without granulomatosis or neoplastic involvement. All four of these criteria must be met for the diagnosis of DILS. A difference in the T-cell receptor (TCR) beta-chains was also revealed between SS patients and subjects with DILS [81]. In a cohort study in Greece, SS-like syndrome (SLS) was confirmed in 7.79% of HIV-positive subjects, in whom CD8^+^ T cell-dominant infiltration was observed in salivary gland biopsy specimens without anti-Ro/SS-A, La/SS-B antibody [82]. An assessment of the prevalence of DILS at an HIV outpatient clinic in the U.S. revealed that 3% of the HIV-seropositive patients (15/523) had DILS with enlargement of the parotid gland and sicca symptoms [83]. In their study of HIV-associated antigen, Yamano et al. observed that 33% of the sera and 47% of the salivary gland specimens from SS patients reacted with p24 (gag) of HIV, although target genes for HIV were not detected in either type of samples from SS patients [84]. Their findings suggest the involvement not of HIV but of an unknown retrovirus in SS.

The presence of human retrovirus-5 (HRV-5) in salivary glands was examined by nested PCR in 55 SS patients and 37 non-SS subjects, and no association of HRV-5 with SS was observed [85]. The estimated prevalence of SLS among 131 HIV patients under an intensive anti-retroviral strategy, i.e., highly active anti-retroviral therapy (HAART), was 7.8% [86]. As of 2020, the number of publications concerning a relationship between SS and HIV has decreased, probably because of the development of HAART and the 2002 description of HIV infection as well as hepatitis C infection in the exclusion criteria in the classification of SS provided by the American-European Consensus Group (AECG) [87].

### 4.2. Clinical and Epidemiological Findings of HTLV-1 Infection in SS

In parallel with investigations of involvement of the HIV in Sjögren’s syndrome, the engagement of HTLV-1 in SS has been researched. In this section, we summarize the clinical and epidemiological studies of the association between HTLV-1 infection and SS. HTLV-1, a member of the type C delta-retrovirus family [88], is a causative agent of adult T-cell leukemia (ATL), which is a hematological neoplastic disorder first described by Takatsuki et al. in 1977 [89]. After HTLV-1 was recognized as causative virus for ATL, the whole base sequence [90] and the function of Tax protein [91] were determined. HTLV-1 is also a causative agent for the tropical spastic paraparesis/HTLV-1-associated myelopathy (HAM) identified in the Caribbean basin and the southeast area of Japan; patients with HAM exhibit slowly progressive neurological symptoms such as spastic gait and bladder and rectal disturbance [92,93]. HTLV-1-associated uveitis [94] is also observed in HTLV-1 carriers, and the frequent complication of Grave’s disease is reported [95]. Broncho-alveolitis is an example of organ involvement in HTLV-1 infection as a pulmonary complication [96]. As transmission pathways of HTLV-1, maternal infection with various levels of HTLV-1 proviral DNA during pregnancy was reported [97]. Representative publications about the detection of genes and proteins in HTLV-1-associated SS are listed in Table 3.

Although several case reports regarding SS associated with HTLV-1 infection are available, it was initially reported that HTLV-1 carriers with SS showed high frequencies of extra-glandular manifestations including uveitis and myopathy [112]. It was subsequently demonstrated that in Nagasaki, Japan (an endemic area for HTLV-1), the prevalence of HTLV-1 in SS patients was significantly higher than that of 27,284 blood donors [113]. In addition, a serum antibody toward HTLV-1 in HTLV-1-seropositive SS patients was similar to that from patients with HTLV-1-associated myelopathy (HAM). In a study conducted in Chile, among 48 patients with HAM, 29.1% of the patients who lacked autoantibodies including anti-Ro/SS-A antibody or rheumatoid factor (RF) showed chronic dacryosialadenitis without being classified as having SS [110]. We observed that 60% of a series of HAM patients fulfilled the preliminary European Community criteria for SS with positive mononuclear cell infiltration in LSGs [114], and these HAM patients with SS showed low frequencies of anti-Ro/SS-A antibody, anti-nuclear antibody, and RF compared to those observed in typical SS.

The high prevalence of SS in HAM patients was also observed in a later investigation in which chronic sialadenitis and exocrine dysfunction were detected by Saxon test and Schirmer’s test, respectively [115]. Magnetic resonance imaging (MRI) revealed that 92% of HAM patients with SS lacked typical characteristics usually found in SS, although the salivary flow rate of these patients was similar to that of SS patients without HAM [99]. We subsequently documented a low frequency of abnormal findings in HTLV-1-seropositive patients with SS compared to those of HTLV-1-seronegative patients with SS [100], although the aforementioned MRI findings showed much clearer imaging differences than those in sialography. The relationship between abnormality in such images and the pathogenesis of SS is discussed later in this review. With reference to our previous study [88] that showed a high frequency of myopathy in HTLV-1-seropositive SS, Cruz et al. [101] noted that their study conducted in Brazil revealed that 38% of HTLV-1-seropositve subjects had fibromyalgia, and their HTLV-1-seropositive subjects had a high prevalence of rheumatic diseases. We found that among atomic-bomb survivors in Japan, the prevalence of HTLV-1 infection in SS patients was higher than that in non-SS subjects [102].

We also re-evaluated the complications of SS experienced by patients with HAM 18 years after we reported the association [116], because the classification criteria were revised as AECG criteria [87] and the aging of HTLV-1-seropositive subjects with the changes in the composition of the population in Japan was established [117,118]. As a result, 38.5% of our patients with HAM were classified as having SS based on the AECG criteria, and the prevalence of anti-Ro/SS-A antibody was significantly lower than that in HTLV-1 carriers complicated with SS. A cross-sectional study performed in Brazil demonstrated that in a group of 272 HTLV-1-seropositive participants at an HTLV-1 clinic, 59 (21.7%) patients (none of whom had HAM) showed sicca symptoms without anti-Ro/SS-A, La/SS-B antibodies [119]. That study also revealed that proinflammatory cytokines were elevated in HTLV-1-seropositive subjects with sicca symptoms regardless of the presence/absence of SS, suggesting that HTLV-1 itself has the potential to induce nonspecific inflammation in salivary glands regardless of the presence of autoimmune disease.

Vale et al. [104] examined the association between HTLV-1 infection and the presence of SS in 129 HTLV-1-seropositive subjects in Brazil; they observed xerostomia and xerophthalmia in 35.7% and 13.95% of the subjects, respectively. In contrast, these HTLV-1-seropositive subjects showed only 0.77% anti-La/SS-B antibody. Regarding pulmonary involvement in SS, we detected an increased frequency of airway diseases in HTLV-1-seropositive SS patients [120], indicating an influence of HTLV-1 infection as a systemic manifestation.

### 4.3. The Detection of HTLV-1 Genes and Proteins in SGs in SS

Similar to the involvement of EBV in SS, the infection of salivary glands by HTLV-1 has also been debated. In 1992, it was reported that the expression of HTLV-1 p19 (a member of the family of gag antigens) was observed in SGECs from 31% of SS patients, 24% of RA patients with SS, and 21% of patients with sicca [105], suggesting the possibility of the presence of an endogenous retrovirus other than the human endogenous retroviral sequence (HRES-1). Mariette et al. [106] reported that *tax* gene (but not *gag*, *pol*, *env*) was detected by both ISH and PCR in labial salivary glands of two of nine patients with SS. They also observed the localization of *tax* in the nuclei of both SGECs and lymphocytes. Sumida et al. [107] later described similar findings with the positive expression of *tax* gene in labial salivary glands from Japanese patients with SS, and they reported that the nucleotide sequence of the *pX* region in labial salivary glands was completely identical to that of the MT-2 cell line, suggesting that the findings of these two studies [106,107] support the concept that at least *tax* gene might be involved in the pathogenesis of SS.

In an aforementioned study conducted in Nagasaki [113], the serum titers of anti-HTLV-1 antibody in patients with HTLV-1-seropositive SS was as high as that in HAM patients, and IgM-class antibodies were frequently detected in the former group. Interestingly, salivary IgA antibodies to HTLV-1 were observed in HTLV-1-seropositive SS patients although a low frequency of these antibodies was observed in the HAM patients and healthy carriers. Four responses to that study [113] arose from France and Austria [108], in which there were similar observations regarding the detection of these antibodies with additional observations in HTLV-1-seropositive SS patients. In these responses, the involvement of *tax* gene and the local synthesis of IgA class anti-HTLV-1 antibody in saliva was discussed. In contrast, a study of salivary glands from 49 patients with SS in Great Britain demonstrated no *tax* expression unlike that of ERV-3, an endogenous human retrovirus [110], suggesting differences in regional characteristics between endemic and non-endemic areas as well as the possibility of PCR contamination.

With respect to HTLV-1-seropositive patients with SS, an investigation using an in situ PCR technique detected HTLV-1 proviral DNA in infiltrating MNCs but not salivary gland epithelial cells of these patients [121]. In contrast, we detected *tax* gene in 18% of the salivary glands of HTLV-1-seronegative SS patients by nested PCR [122], suggesting that the involvement of HTLV-1 in HTLV-1-seronegative patients might be limited because the viral load in salivary glands from HTLV-1-seronegative SS patients was much lower than that from HTLV-1-seropositive patients. A study conducted in France and using ISH detected HTLV-1 RNA in both salivary gland epithelial cells and lymphoid cells of HTLV-1-seronegative SS patients, although *tax* gene was expressed in LSGs from these patients [123]. Sasaki et al. later described the presence of TCR Vbeta gene in infiltrating lymphocytes of salivary glands from HTLV-1-seropositive SS patients, in which specific T cells with Vbeta5.2, 6, and 7 were used, indicating the involvement of an HTLV-1-driven T-cell activation in SS [124].

**Figure 1:**
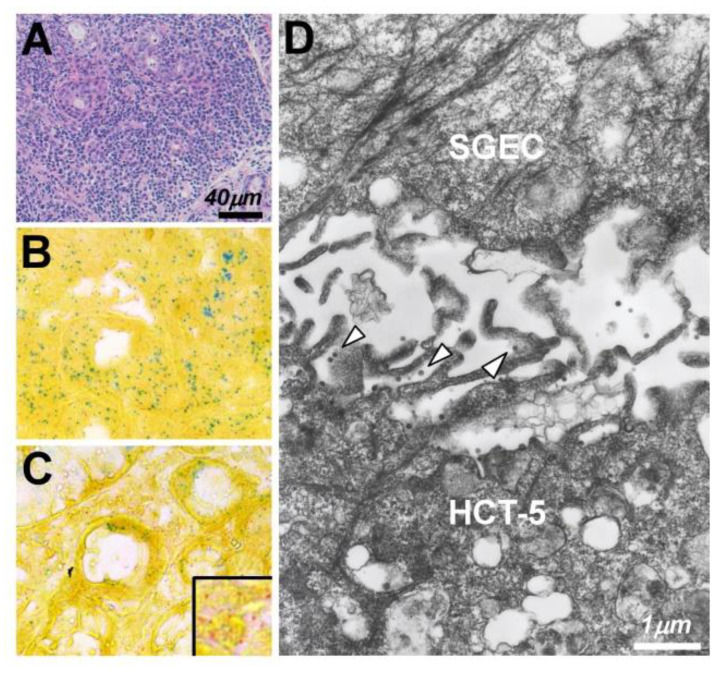
The expression of *tax*/*HBZ* and HTLV-1 virions in salivary glands (SGs) of patients with Sjögren’s syndrome (SS). (**A**) Massive lymphocytic infiltration was by hematoxylin-eosin staining in a labial salivary gland from a patient with sicca symptoms and adult T-cell leukemia (ATL). The expression of *tax*/HTLV-1 bZIP factor (*HBZ*) in SGs from a patient with ATL (**B**) and patients with HTLV-1-associated myelopathy complicated with SS (**C**)**,** examined by in situ hybridization. A dominant expression of *HBZ* (*green*) was observed in the ATL SGs in both infiltrating mononuclear cells (MNCs) and ducts (**B**). In contrast, a dominant expression of *tax* (*red*) was observed in MNCs of salivary glands from patients with HAM-SS (**C**). Electron microscopy (**D**) revealed the existence of HTLV-1 virions (*arrowheads*) at the contact face between HCT-5 cells (an HTLV-1-infected cell line) and salivary gland epithelial cells (SGECs).

As for other inflammatory conditions, Mariette et al. examined the expression of *tax* gene in LSGs from SS patients and patients with chronic inflammatory diseases [125]. They detected *tax* gene in 30% of the SS patients and 28% of the non-SS patients. These results suggested that HTLV-1 may contribute to the development of chronic inflammation regardless of the presence/absence of SS. Although the number of publications concerning HTLV-1-related genes in the salivary glands of individuals with SS has decreased since 2000, a 2012 report from Korea noted that *tax* gene was detected in 3.8% of a series of SS patients, and HTLV-1 p19 or Tax protein was revealed by IHC in LSGs from >40% of the SS patients [126]. With regard to HTLV-1 genes other than the previously reported genes, we examined the expressions of HTLV-1 bZIP factor (*HBZ*) and *tax* gene by ISH, which showed that both genes expressed in infiltrating MNCs and SGECs from HAM-SS patients and a patient with adult T-cell leukemia (ATL), although the expression of *tax* gene was dominant in MNCs of the HAM-SS patients [127] (Figure 1A,B).

*HBZ* gene, which is encoded by a minus strand of the HTLV-1 provirus, is known to induce chronic inflammation in ATL through the expression of Foxp3, and it was suggested that the expression of *HBZ* has the potential to induce Foxp3 gene transcription in CD4^+^ T cells from *HBZ* transgenic mice [111,128]. In accord with these experimental observations, we also observed a high expression of Foxp3 in salivary gland tissue from a patient with ATL and patients with HAM-SS. The involvement of *HBZ* gene in HTLV-1-seropositive SS patients was newly considered after the observed outbreak of chronic inflammation in this population.

### 4.4. The Mode of HTLV-1 Infection in SS Salivary Glands

As a potential mechanism of HTLV-1 transmission to salivary glands, a brief discussion of the concept of HTLV-1 transmission between lymphocytes is presented next. Generally, an efficient HTLV-1 infection requires cell-to-cell contact [98]; cell-free viral transmission is insufficient. HTLV-1 preferentially infects CD4^+^ T cells but has shown a low frequency of infection of CD8^+^ T cells. Two theories and an additional proposal have been published with regard to the cell-to-cell transmission of HTLV-1 virions. Igakura et al. proposed a concept that they named the ‘viral synapse’ which consists of an assembly of HTLV-1 gag protein complex and viral genomes at the microtube-organizing center, with a polarized contact surface between HTLV-1-infected and uninfected cells [103]. Another theory was proposed by Pais-Correia and colleagues, who demonstrated that extracellular matrix and linker structures including collagen, agrin, tetherin, and galectin-3 are involved in the delivery of the HTLV-1 viral particles at the contact surface [129,130]. In this system, a biofilm-like viral assembly with extracellular components on the HTLV-1-infected CD4^+^ T cell contacts an uninfected CD4^+^ T cell, resulting in the transmission of virions by cell-to-cell contact.

**Figure 2:**
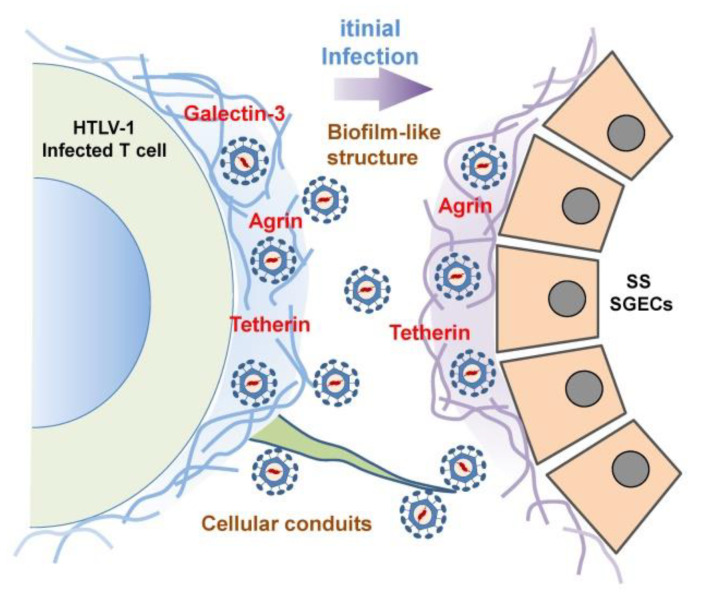
A hypothetical scheme of the initial transmission of HTLV-1 virions. HTLV-1 virions exist with extracellular matrix proteins or linker proteins including galectin-3, agrin, and tetherin. After the initial contact of HCT-5 cells with SGECs, HTLV-1 virions are Table 1. virions by the extension of a long structure that is stretched from the surface of HCT-5 cells.

As an additional system to convey HTLV-1 virions from infected to uninfected CD4^+^ T cells, the concept of a cellular conduit was described by Van Prooyen et al. [131]. They showed that cellular conduits that expressed HTLV-1 p8 protein with an accumulation of lymphocyte function-associated antigen (LFA-1) acted as an intermediary with respect to the transmission of HTLV-1 virions. This novel concept was introduced as a third pathway for the transmission of HTLV-1 virions in concert with the function of p8 accessory protein [132]. In coming to detection of the initial dynamic state of HTLV-1 infection of salivary glands, we investigated the involvement of these three pathways in an in vitro study as a single publication [133] by using an HTLV-1-infected cell line, HCT-5. Although agrin, tetherin, and galectin-3 were expressed on the surface of HCT-5 cells, agrin and tetherin were observed with HTLV-1 Gag-positive particles on cultured SGECs after a co-culture with HCT-5 cells (Figure 1C). We observed no formation of virological synapses between HCT-5 cells and SGECS, although an immunofluorescence analysis suggested the possibility of the involvement of a cellular conduit (Figure 2).

The handing over of HTLV-1 virions between HTLV-1-infected cells and SGECs was confirmed by electron microscopy, although we found no endogenous virions on SGECs after a co-culture with an uninfected T-cell line.

### 4.5. Immunological Modulation of HTLV-1 Infection in SS

The association between the pathogenesis of SS and HTLV-1 infection has been addressed with experimental approaches including in vitro studies, animal studies, and studies with human tissue samples including salivary gland tissue. As a catalyst of experimental studies, Green et al. developed HTLV-1 *tax* gene transgenic (Tg) mice [134]. Interestingly, the *tax* Tg mice showed human SS-like pathology including cellular infiltration as well as a proliferation of ductal epithelial cells. It was also demonstrated that the introduction of *env-pX* gene to Wistar-King-Aptekman-Hokudai (WKAH) rats induced human SS-like characteristics such as chronic sialadenitis and dacryoadenitis as well as arthritis, necrotizing arteritis, polymyositis, myocarditis and dermatitis with elevated RF [135]. These characteristics in *env-pX* rats showed a direct involvement of HTLV-1 infection in the induction of autoimmunity.

Concerning the involvement of HTLV-1 infection in WKAH rats, Yoshiki et al. showed that one of their WKAH rats that possessed the RT1k haplotype presented neurological symptoms resembling HAM [136], suggesting that differences in the HTLV-1 haplotype might influence the clinical phenotypes. With respect to the involvement of HTLV-1 in apoptosis, we examined the frequency of terminal deoxynucleotidyl transferase-mediated dUTP nick-end labeling (TUNEL) staining [137] and Fas/Fas ligand (FasL) [138,139] staining of HTLV-1-seropositive and -seronegative SS patients because Fas/FasL system-mediated apoptosis has been shown to play a role in the pathogenesis of many autoimmune diseases [140]. Although we observed a low frequency of TUNEL-positive apoptosis in both SGECs and infiltrating MNCs, there was no significant between-cell type difference regardless of the presence/absence of anti-HTLV-1 antibody.

In our study of the involvement of anti-apoptotic molecules in HTLV-1-seropositive SS patients, we investigated the expression of Bcl-2 family proteins in PBMCs and LSGs from SS patients [141], because HTLV-1-mediated bcl-2 family proteins inhibited the apoptosis of T-cell lines including JPX-9 cells (a subline of Jurkat human T cells) and a mouse T-cell line (CTLL-2) through the activation of nuclear factor kappa B [142,143]. We observed, that compared to the Bax expression in the LSGs and PBMCs, there was a dominant expression of Bcl-2 as well as CD40/CD40 ligand in the LSGs regardless of HTLV-1 infection.

We subsequently detected members of the mitogen-activated protein kinase (MAPK) superfamily [144] including c-Jun N-terminal kinase and p38 that were accompanied by CD40 or X-chromosome-linked inhibitor of apoptosis protein (XIAP) [145] in salivary gland tissue from SS patients, but the differences in expression of XIAP were not determined by HTLV-1 infection. We then focused on the characteristics of the imaging of glands from HTLV-1-seropositive SS patients [100,101] and germinal centers (GCs) because it is known that GCs are a specific site for B-cell activation and selection [146,147]. Notably, Amft et al. showed that lymphoid tissue-homing chemokine B cell-attracting chemokine (BCA-1, also known as C-X-C motif chemokine ligand 13, or CXCL13) was specifically expressed in the GC structure and endothelial cells in salivary glands from SS patients. CXCL13 had the effect of specifically attracting human B cells that expressed BLR1, which was later named CXCR5 [109]. We observed a low number of GCs as well as a low expression of CXCL13 in HTLV-1-seropositive SS cases, and the HAM-SS cases in particular lacked GC formation, suggesting that HTLV-1 infection might inhibit the formation of GCs and the subsequent selection of high-affinity B cells that develop into plasma cells.

Concerning chronic lymphocytic infiltration in salivary glands without specific autoantibodies in SS, the DILS observed in salivary glands of individuals affected by HIV with the infiltration of CD8^+^ T cells [148] is a reminder that the dominant infiltration of CD4^+^ T cells in the salivary glands of HAM-SS patients with hypergammaglobulinemia resembles that observed in DILS. In addition, we treated an ATL patient with bilateral parotid gland enlargement, xerostomia, and xerophthalmia [149], and although this patient showed a massive infiltration of CD4^+^ T cell-dominant lymphocytes, antibodies against Ro/SS-A, La/SS-B were negative. There are differences in the phenotypes of infiltrating T cells, but the existence of DILS in HIV-positive patients and a patient with ATL suggests the possibility that a massive infiltration of T cells with sicca symptoms would fulfill the classification criteria of SS [87,150].

Lastly, we discuss the immunological alterations observed in that area after the integration of HTLV-1 into host non-lymphoid cells including SGECs. HTLV-1 infection confirmed by semiquantitative PCR or ELISA was observed in 2.6% of retinal glial cells along with the production of inflammatory cytokines including IL-6 and TNF-α [151]. A similar in vitro study using synovial cells showed that a co-culture of synovial cells with HTLV-1-infected T-cell lines led to the expression of HTLV-1 core antigen and HTLV-1 proviral DNA on the synovial cells along with the production of granulocyte/macrophage colony-stimulating factor [152]. Interestingly, Carvalho Barros et al. demonstrated that HTLV-1 infected thymic epithelial cells (TECs), and the TECs had the potential to convey HTLV-1 virions to CD4^+^ T cells [153].

Regarding the HTLV-1 infection of SGECs from SS patients, we confirmed HTLV-1 proviral DNA and proteins by in situ PCR and immunofluorescence, respectively after co-culture with an HTLV-1-infected T-cell line [154]; in addition, the concentrations of soluble intracellular adhesion molecule (ICAM)-1 and RANTES (regulated on activation, normal T cell expressed and secreted) and IFN-γ-inducible 10-kD protein (IP)-10 increased after the co-culture of SGECs from SS patients with HCT-5 cells. It was striking that the cytoplasmic expression of ICAM-1, CXCL1, RANTES, IL-8, and IP-10 on SGECs was also augmented after co-culture. Our findings indicated that HTLV-1 had the capability to infect SGECs, and these SGECs also expressed inflammatory cytokines or chemokines. However, these phenomena are currently limited to in vitro findings.

## 5. Overview of the Involvement of Viruses in SS

**Figure 3:**
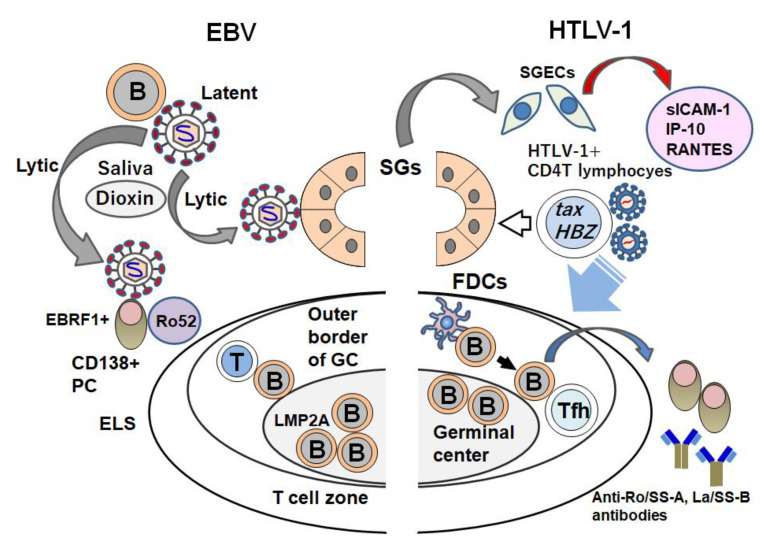
The different roles of EBV and HTLV-1 in the pathogenesis of SS. EBV exists in B cells and/or B cells that expresses LMP2A in ectopic lymphoid structures (ELSs) as a latent phase. Once the reactivation of EBV is induced by environmental factors (including dioxin), EBV changes to the lytic phase. Lytic EBV can infect SGECs and perifollicular EBRF^+^ plasma cells (PCs) that can react with Ro52. In contrast, HTLV-1 that bears *tax* and HTLV-1 bZIP factor (*HBZ*) infects salivary glands of patients with SS. An in vitro study showed that after the HTLV-1 infection of salivary glands, SGECs express inflammatory cytokines or chemokines including soluble ICAM-1, IP-10, and RANTES. Inhibitory effects of HTLV-1 toward ELS components (including B cells), PCs that produce anti-Ro/SS-A, La/SS-B antibodies, follicular dendritic cells (FDCs), and follicular helper T cells (Tfh) have been considered.

We have outlined the involvement of viruses (centering on EBV and HTLV-1 as environmental factors) in the pathogenesis of SS (Figure 3). These two viruses have distinct directionalities in the phenotypes of lymphocytes with respect to the formation of clinical characteristics (including exocrine dysfunction) in SS. The existence of conflicting findings with regard to the involvement of viruses in the development of SS must be considered, but it is anticipated that these viruses also have some unique actions in the formation of ELSs, i.e., ectopic GCs and the autoantibody production system.

EBV showed robust affinity to the ELS, whereas HTLV-1 infection might inhibit the formation of the ELS in accord with its proviral load (PVL). In addition, EBV has high affinity to B cells or plasma cells (PCs) in accord with the alteration of its phase from the latent mode to the lytic mode, which is suggested to be stimulated by environmental substances. However, SS patients with HAM who show higher PVLs than HTLV-1 carriers have a low frequency of the autoantibodies that are found in typical SS patients. Regarding the HTLV-1-mediated induction of SS, various theories have attempted to explain the pathogenesis of SS. A definite epidemiological relationship between HTLV-1 infection and SS has been established by the findings obtained in *tax* or *env-pX* Tg animal models and in vitro studies. However, unlike EBV infection, in HTLV-1-infected subjects there is no direct evidence indicating molecular biological effects on the production of autoantibodies in SS patients.

To address this scarcity of evidence, it is necessary to determine whether HTLV-1 infection has immunological influences on specific autoantibody production systems. In other words, it should be clarified whether or not HTLV-1 simply modulates the pathogenesis of existing typical SS. For example, HTLV-1 might influence immune cells including B cells, PCs, and/or T follicular helper cells in the ELS to reduce autoantibody production. It is also possible that CD4^+^ T cells mobilized by HTLV-1 infection simply form an aggregation of DILS-like lymphocytes as observed in HIV infection or the sialadenitis observed in ATL. Unlike HIV infection with sialadenitis, a great mass of HTLV-1 infection occurs by maternal infection; in contrast, SS usually develops in middle age. Clinicians should thus be concerned about the effects and or the immunological modulation of HTLV-1 infection during the 40–50 years before the development of SS.

In cases of EBV infection, it is suspected that reactivation in the lytic phase promotes immunological dysfunction. However, when HTLV-1 infection occurs by maternal infection, HTLV-1 that shows no change of infective form continues to infect CD4^+^ T cells for several decades. Investigations that may determine the triggers of the formation of SS-like sialadenitis in salivary glands over a prolonged period are further research tasks that will also elucidate the differences in engagement between EBV and HTLV-1.

## 6. Conclusions

Molecular biological approaches have enhanced our understanding of the contribution of Epstein-Barr virus to the pathogenesis of Sjögren’s syndrome. The involvement of HTLV-1 has been thought to vary among endemic areas, whereas EBV infection is ubiquitously observed regardless of regional characteristics. In addition, the aging of HTLV-1-infected populations might influence the prevalence of HTLV-1-seropositive SS. To determine the direct contribution of both EBV and HTLV-1 in the pathogenesis of SS, the integration of epidemiological and experimental studies including virological and molecular immunological approaches is strongly desired.

## Figures and Tables

**Table 1:** Representative publications suggesting involvement of Epstein-Barr virus (EBV) infection in patients with Sjögren’s syndrome.

Authors [Ref.]	Origin	Year	Detection	Key Points
Venables [40]	UK	1985	Indirect IF	IgG antibody against EBVCA was detected in sicca syndrome patients, RA patients, and healthy subjects.
Fox [41]	USA	1986	Immunostaining, IB, slot hybridization	EA-D was detected in 8 of 14 SGs and EBVDNA was detected in 8 of 20 saliva samples from SS patients.
Yamaoka [42]	Japan	1988	Indirect IF	IgG and IgM antibodies against EBV capsid antigen and EBV excretion from the oropharynx were observed in SS patients.
Saito [43]	USA	1989	PCR	The usefulness of PCR was shown, revealing viral DNA in SG epithelial cells from SS patients.
Venables [44]	UK	1989	ISH, IF, immunostaining	EBVDNA was detected in SGs from 60% of healthy subjects and 17% of SS patients.
Mariette [45]	France	1991	ISH, PCR	A combination of ISH and PCR was introduced, showing a high positive rate in patients with SS.
Inoue [46]	Japan	1991	ELISA, IB	Elevated IgG antibody against EBNA antigens was detected in sera from SS patients.
Fox [45]	USA	1989	RFLP	Two SS patients with B-cell lymphoma showed an unusual RFLP pattern.
Tateishi [47]	Japan	1993	FCM, PCR	Massive EBV production was confirmed in an SS PBMC-derived B-cell line.
Newkirk [48]	Canada	1996	ELISA	Antibodies against early EBV peptides such as BHRF1 were detected in SS patients.
Merne [49]	Finland	1996	ISH, PCR	ISH detected EBVDNA in SGs from 19% of SS patients and 3% of controls.
Inoue [50]	Japan	2001	IF, RT-PCR, IB	ZEBRA mRNA in SGs from SS patients was observed, and EBV-activated lymphocyte mediated the production of 120-kDa alpha-fodrin.
Inoue [51]	Japan	2012	Luciferase assay	With the use of saliva from SS patients, dioxin augmented the transcription of BZLF1that stimulated conversion to the lytic phase in EBV.
Pasoto [52]	Brazil	2013	ELISA	Frequent anti-EA-D antibodies were observed in SS patients.
Croia [53]	UK	2014	RT-PCR, ISH, immunostaining	Affinity of lytic phase EBV toward PCs in ELS was shown. Perifollicular PCs showed reactivity toward Ro52.

EA-D: EBV-encoded early antigen, ELS: ectopic lymphoid structure, FCM: flow cytometry, IF: immunofluorescence, IB: immunoblot, ISH: in situ hybridization, PBMC: peripheral blood mononuclear cell, PCR: polymerase chain reaction, PC: plasma cell, RA: rheumatoid arthritis, RFLP: restriction fragment length polymorphism, RT: reverse transcription, SG: salivary gland.

**Table 2:** Representative publications suggesting involvement of retroviruses (other than HTLV-1) with Sjögren’s syndrome.

Author [Ref. No]	Origin	Year	Findings
Schiødt [75]	USA	1989	9 patients with HIV-SGD showed parotid gland enlargement and 11 patients with xerostomia. CD8 T cells showed in LSGs. No EBV and CMV was detected.
Garry [76]	USA	1990	Particles of human intracisternal A-type retrovirus that were similar to HIV were observed in LSGs of SS patients.
Talal [77,78]	USA	1990	Antibodies against HIV-1 were detected in 30% of SS patients and 1 among 120 normal subjects. Seropositive SS patients reacted p24 (gag) only.
Itescu [79]	USA	1990	Among 17 HIV patients showed, 14 patients had xerostomia and 11 patients had abnormal Gallium uptake. CD8 cells in LSGs and HLA-DR5 were associated with these patients.
Itescu [80]	USA	1991	Concept of diffuse infiltrative lymphocytosis syndrome (DILS) in patients with HIV was released. CD8 T cell infiltration and involvement of exocrine glands and other tissues were shown.
Dwyer [81]	USA	1993	Sharing of the sequences of 59 beta-chains in 5 DILS patients was shown. Additionally, appearance of certain common V beta and J beta gene segments was found. Usage of TCR beta chains was different from primary SS patients.
Kordossis [82]	Greece	1998	The prevalence of SLS in HIV-1-positive patients was 7.79%, in which 6 out of 14 patients showed sialadenitis with CD8 T cell dominancy. These patients had no anti-Ro/SS-A and La/SS-B antibodies but had hypergammaglobulinemia.
Williams [83]	USA	1998	Among 523 patients with HIV, 15 patients (3%) showed DILS with racial differences. DILS patients had high CD8 counts and clinical stage of DILS patients was less than non-DILS patients.
Yamano S [84]	Japan	1997	Sera from 33% SS patients reacted against p24 (gag) and LSGs from 47% SS patients reacted against anti-p24 monoclonal antibody. Additionally, A-type-like retroviral particles were detected by electron microscopy in LSGs from SS patients.
Rigby [85]	UK	1997	By nested PCR, 1 non-SS patient and 1 secondary SS patient showed positive for human retrovirus-5 (HRV-5) among 92 samples. Three different sequences of HRV-5 were 98% identical to originally detected sequence.
Panayiotakopoulos [86]	Greece	2001	Reduced prevalence of SLS after introduction of the highly active anti-retroviral therapy (HAART) was detected with only 2 positive findings among 17 SGs biopsy. Prevalence of 7.8% in pre-HAART period disappeared after execution of HAART.

CMV: cytomegalovirus, ELS; ectopic lymphoid structures, HIV: human immunodeficiency, HRV: human retrovirus, PCR; polymerase chain reaction, SGD: salivary gland disease, SGs; salivary glands, SLS: Sjögren’s -like syndrome, TCR: T cell receptor.

**Table 3:** Detection of HTLV-1-related genes and proteins in sera or SGs from patients with Sjögren’s syndrome.

Authors [Ref.]	Origin	Year	Detection	Key Points
Green [98]	USA	1989	IB, IHC	Tax protein expression was detected in SGs and muscle of *tax* transgenic mice as a first mouse model of SS.
Shattles [99]	UK	1992	Indirect IF	HTLV-1 p19 was detected in 31% of epithelial cytoplasm of SS patients by using a monoclonal antibody specific for retrovirus.
Mariette [100]	France	1993	ISH, PCR	*tax* gene only was detected in 2/9 LSGs from SS patients, although these patients did not react to anti-HTLV-1 antibodies.
Sumida [101]	Japan	1994	RT-PCR	*tax* gene only was detected in 4 of 14 LSGs from Japanese SS patients. The *pX*IV region sequence had homology to MT-2 cells.
Terada [102]	Japan	1994	IB, PCR	Salivary IgA antibodies to SS patients with anti-HTLV-1 antibody and high prevalence of HTLV-1 in SS patients were observed.
Yamazaki [103]	Japan	1997	RT-PCR	Transgenic rats with env-pX gene showed chronic sialadenitis as well as arthritis, vasculitis, and polymyositis.
Ohyama [104]	Japan	1998	PCR, in situ PCR hybridization	Extracted DNA from HTLV-1 seropositive SS contained full proviral DNA. Infiltrating T cells had proviral DNA in the nucleus.
Tangy [105]	France	1999	PCR, ISH	Expression of tax was observed in SGs of HTLV-1-seropositive SS and HTLV-1-seronegative SS patients and sicca subjects.
Sasaki [106]	Japan	2000	PCR, SSCP, sequencing of cDNA	Use of TCR Vβ5.2, 6, and 7 in LSGs from HTLV-1-seropositive SS patients. Vβ7 with conserved AA motif was observed in these patients.
Mariette [107]	France	2000	PCR	*tax* gene was detected in LSGs from 30% of SS patients, 28% of patients with inflammatory diseases, and 4% of healthy subjects.
Lee [108]	Korea	2012	PCR, nested PCR, IHC	*tax* gene but not *pX*, *p19* or *pol* was observed in PBMCs from 3.8% of SS patients. p19 and Tax were expressed in LSGs.
Nakamura [109]	Japan	2015	*In situ* PCR, IF	Expressions of proviral DNA, Gag, and chemokines were observed on SGECs co-cultured with a HTLV-1-positive cell line.
Nakamura [110]	Japan	2018	Real-time PCR, ISH, IHC	In LSGs of HAM-SS, dominant *tax* and *HBZ* was observed with the expression of Foxp3 in LSGs from HAM-SS and ATL patients.
Nakamura [111]	Japan	2019	IF, EM	A biofilm-like structure but not virus synapses was involved in the transmission of HTLV-1 virions from HTLV-1-positive cells to SGECs.

AA: amino acid, EM: electron microscopy, HAM: HTLV-1-associated myelopathy, IF: immunofluorescence, IB: immunoblot, IHC: immunohistochemistry, ISH: in situ hybridization, LSG: labial salivary gland, PBMC: peripheral blood mononuclear cell, PCR: polymerase chain reaction, RA: rheumatoid arthritis, RT: reverse transcription, SG: salivary gland, SGEC: salivary gland epithelial cell, SSCP: single-strand confirmation polymorphism, TCR; T-cell receptor.

## Abbreviations

AhR	aryl hydrocarbon receptor
AID	autoimmune disease
APS	anti-phospholipid syndrome
ATL	adult T-cell leukemia
BCA	B cell-attracting
BLEL	benign lymphoepithelial lesion
CAEBV	chronic active EBV
CMV	cytomegalovirus
CXCL	C-X-C motif chemokine ligand
DILS	diffuse infiltrative lymphocytosis syndrome
EA	early antigen
EBER	EB encoding region
EBNA	EB nuclear antigen
EBV	Epstein-Barr virus
EBVCA	EBV capsid antigen
ELISA	enzyme-linked immunosorbent assay
ELS	ectopic lymphoid structures
ESSDAI	EULAR Sjögren’s Syndrome Disease Activity Index
FasL	Fas ligand
GC	germinal center
GBS	Guillain-Barré Syndrome
HAART	highly active anti-retroviral therapy
HHV	human herpes virus
HIV	human immunodeficiency virus
HLA	human leukocyte antigen
HRV	human retrovirus
HTLV-1	human T-cell leukemia virus type 1
HRES	human endogenous retroviral sequence
ICAM	intracellular adhesion molecule
IFN	interferon
IHC	immunohistochemistry
IL	interleukin
IP	interferon-γ-inducible 10-kD protein
IRF	IFN-regulatory factor
ISH	in situ hybridization
LFA	lymphocyte function-associated antigen
LG	lacrimal gland
LSG	labial salivary gland
LMP	latent membrane protein
LPD	lymphoproliferative disorder
MALT	mucosa-associated lymphoid tissues
MAPK	mitogen activated protein kinase
MDA	melanoma differentiation associated gene
MHC	major histocompatibility complex
MNC	mononuclear cell
MTX	methotrexate
MZL	marginal zone lymphoma
NHL	non-Hodgkin’s lymphoma
NOD	nucleotide-binding oligomerization domain
NLR	(NOD)-like receptor
PAMP	pathogen-associated molecular pattern
pDC	plasmatoid dendritic cell
PCR	polymerase chain reaction
PCs	plasma cells
PVL	proviral load
RA	rheumatoid arthritis
RANTES	regulated on activation, normal T cell expressed and secreted
RF	rheumatoid factor
RIG-I	retinoic acid inducible gene-I
RLR	RIG-I-like receptor
RNA	ribonucleic acid
SG	salivary gland
SGEC	SG epithelial cell
SLE	systemic lupus erythematosus
SLS	SS-like syndrome
SS	Sjögren’s syndrome
TCDD	tetrachlorodibenzo-p-dioxin
TCR	T-cell receptor
TEC	thymic epithelial cell
Tg	transgenic
TLR	toll-like receptor
TNF	tumor necrosis factor
TRX	thioredoxin
TUNEL	terminal deoxynucleotidyl transferase-mediated dUTP nick-end labeling
XIAP	X-chromosome-linked inhibitor of apoptosis protein
WKAH	Wistar-King-Aptekman-Hokudai
